# Pre-gelation staining expansion microscopy for visualisation of the *Plasmodium* liver stage

**DOI:** 10.1242/jcs.261377

**Published:** 2023-11-30

**Authors:** Kodzo Atchou, Bianca Manuela Berger, Volker Heussler, Torsten Ochsenreiter

**Affiliations:** ^1^ Institute of Cell Biology, University of Bern, 3012 Bern, Switzerland; ^2^ Graduate School for Cellular and Biomedical Sciences, University of Bern, 3012 Bern, Switzerland

**Keywords:** *Plasmodium*, Malaria, Expansion microscopy, Mitochondria, Lysosomes

## Abstract

Fluorescence and light microscopy are important tools in the history of natural science. However, the resolution of microscopes is limited by the diffraction of light. One possible method to circumvent this physical restriction is the recently developed expansion microscopy (ExM). However, the original ultrastructure ExM (U-ExM) protocol is very time-consuming, and some epitopes are lost during the process. In this study, we developed a shortened pre-gelation staining ExM (PS-ExM) protocol and tested it to investigate the *Plasmodium* liver stage. The protocol presented in this study allows expanding of pre-stained samples, which results in shorter incubation times, better preservation of some epitopes and the advantage that non-expanded controls can be performed alongside using the same staining protocol. The protocol applicability was accessed throughout the *Plasmodium* liver stage, showing isotropic five-fold expansion. Furthermore, we used PS-ExM to visualise parasite mitochondria as well as the association of lysosomes to the parasitophorous vacuole membrane (PVM) as an example of visualising host–pathogen interaction. We are convinced that this new tool will be helpful for a deeper understanding of the biology of the *Plasmodium* liver stage.

## Teaser

**Summary:** A protocol for investigating the *Plasmodium* liver stage that involves the expansion of pre-stained samples, leading to a significantly expedited procedure, and enhanced preservation of specific epitopes.

## INTRODUCTION

Malaria is a debilitating and potentially fatal disease caused by *Plasmodium* parasites. It is a major global health issue, affecting millions of people in developing countries, particularly in sub-Saharan Africa (Snow et al., 2005; WHO, 2021, https://www.who.int/publications/i/item/9789240040496). Despite extensive research efforts made, malaria remains a major public health challenge, and new treatment options are needed to effectively control and prevent the disease (Tse et al., 2019). A major challenge in the development of new effective anti-malaria drugs is the ability of the parasite to rapidly develop resistance to antimalarial drugs (Plowe, 2022; White, 2004). The *Plasmodium* life cycle is complex, consisting of both asexual and sexual stages that are present in the vertebrate host and in the mosquito. The transmission of malaria occurs when an infected female *Anopheles* mosquito bites a human and releases up to several 100 sporozoites into the skin (Amino et al., 2006; Beier, 1998; Venugopal et al., 2020). Sporozoites are motile and a proportion of them enter blood vessels and are transported with the blood flow to the liver where they invade hepatocytes (Tavares et al., 2013). Inside the liver cells, the parasite resides within a parasitophorous vacuole (PV), in which the parasite proliferates from one single sporozoite to thousands of daughter parasites, called merozoites. This process is also known as schizogony. The merozoites exit the liver cells in vesicles known as merosomes that are transported to adjacent blood vessels (Mota et al., 2001). When the merosomes rupture, they release the merozoites, which subsequently invade erythrocytes. The liver stage represents a crucial opportunity for anti-malaria interventions as it is clinically silent. The characteristic symptoms of malaria only occur upon repeated rounds of asexual blood stage replication and the associated rupture of the red blood cell (Derbyshire et al., 2011; Vaughan and Kappe, 2017). In the infected red blood cells, some merozoites develop into gametocytes (Cowman and Crabb, 2006). Gametocytes are the sexual form of the parasite which, when taken up during the blood meal of a mosquito, differentiate into gametes and mate in the midgut of the insect to form a zygote. This motile parasite stage transmigrates the midgut epithelium and forms a so-called oocyst, inside which sporozoites develop. Upon rupture of the oocyst, sporozoites migrate to the salivary gland of the mosquito and can be transmitted to the next human host. An important tool for the discovery of new treatment options is a better understanding of molecular details of the life cycle of the parasite and host–pathogen interactions. Conventional light microscopy allowed us to gain insight into cellular organisation (Rankin et al., 2010). However, due to the diffraction of light, the maximal resolution lies between 200–300 nm (Heintzmann and Ficz, 2006). Subcellular imaging of the parasite therefore remains challenging and requires super-resolution techniques, such as photo-activated localisation microscopy (PALM), stochastic optical reconstruction microscopy (STORM), structured illumination microscopy (SIM), and stimulated emission depletion (STED) microscopy (reviewed by Heintzmann and Ficz, 2006). However, they require expensive and complex microscopical equipment and special sample preparation (Heintzmann and Ficz, 2006). In comparison, the recently developed expansion microscopy (ExM) is relatively straightforward and does not require complicated preparations or specialised equipment, making it accessible to researchers all over the world (Chen et al., 2015; Gambarotto et al., 2019; Gao et al., 2017). In a relatively short period of time, ExM became a new cutting-edge imaging technique that has revolutionised the field of microscopy. The physical expansion of the sample results in a significant improvement in spatial resolution compared to traditional light microscopy techniques. Expansion microscopy is based on the usage of a hydrogel matrix. The hydrogel matrix is composed of a polymer network that is covalently attached to the biological sample and expands upon hydration (Chen et al., 2015). There are several different expansion microscopy protocols that have been developed, each with its own strengths and limitations (Chen et al., 2015; Gambarotto et al., 2021; Tillberg et al., 2016). The original ExM protocol developed by Edward Boyden's group in 2015 has been widely used and has been modified and improved over the years to increase the accuracy and specificity of the technique (Chen et al., 2015; Gao et al., 2017). Nevertheless, the basic principles of the protocol remain the same. One of the currently most widely used expansion microscopy protocols is ultrastructure expansion microscopy (U-ExM), which has been shown to preserve the cellular architecture in a near-native manner, achieving a four-fold expansion (Gambarotto et al., 2019, 2021). In comparison to the original protocol from Edward Boyden's group, the staining is performed after (instead of before) embedding the sample in the swellable gel. The post-expansion staining reduces antibody competition through the decrowding of the epitopes and prevents fluorophore loss (Gambarotto et al., 2019). However, the protocol is time-consuming, requiring roughly 2 days. Given that the antibody staining is performed on the gel, the incubation times are rather long with ∼3 h for each antibody incubation (Gambarotto et al., 2021). Another limitation is the loss of epitopes during the denaturation step. An alternative to the post-expansion labelling protocol is the proExM protocol developed by the group of Joshua Vaughan (Chozinski et al., 2016). Here, biological samples are stained with fluorescent antibodies before the gelation process begins. The fluorescent labels are afterwards covalently linked to the polymer. Subsequent protease treatment is used for exposing protein epitopes while preserving the signal of the fluorescent labels, which are fairly protease resistant. This approach has the advantage that existing stained samples can be entered into the expansion process and non-expanded samples can be performed under the same conditions (Chozinski et al., 2016). Since its first application in 2015, ExM has been applied to a variety of samples, including cells, tissues and even entire organisms (Chen et al., 2015; Gao et al., 2017). Recently, the U-ExM was used to study the molecular architecture of *Plasmodium* gametocytes (Chozinski et al., 2016; Rashpa and Brochet, 2022). Furthermore, U-ExM allowed insight into the asexual *Plasmodium* blood stage to be gained (Liffner and Absalon, 2021). The aim of the present study was to optimise the U-ExM protocol developed by Gambarotto et al. to study the *Plasmodium* liver stage (Gambarotto et al., 2019, 2021). With some major adaptations for pre-stained samples, we used this method to visualise different developmental stages of the *Plasmodium* liver stage. This fast and easy protocol will now allow researchers to gain a more detailed insight into the *Plasmodium* liver stage.

## RESULTS

### Five-fold isotropic expansion of *Plasmodium* liver stage with pre-gelation staining expansion microscopy

The aim of this study was to adapt the U-ExM protocol to investigate intracellular *Plasmodium* parasites during the liver stage development. The protocol developed by Gambarotto et al. in 2019 results in a near-native expansion of cultured cells (Gambarotto et al., 2019, 2021). In the original protocol, the antibody staining was performed after embedding the cells in a swellable gel, which resulted in a rather long antibody incubation time of ∼3 h. Furthermore, some epitopes were lost during the denaturation prior to the antibody staining. Additionally, the non-expanded controls had to be prepared separately. In this study, the original U-ExM protocol was modified to allow expansion of pre-stained specimens (pre-gelation staining ExM; PS-ExM). Overall, the reagents used for fixation (formaldehyde), anchoring (acrylamide), gelation and denaturation were identical to the ones previously described for U-ExM (Gambarotto et al., 2019, 2021). However, the incubation time for each of the steps was strongly reduced to better preserve the fluorescent signal. A comparison between the original and the adapted PS-ExM protocol version is shown in Table 1.

**Table 1.**
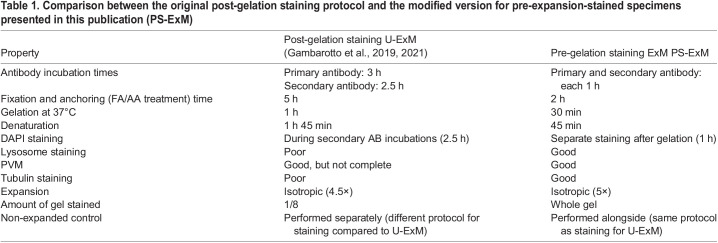
Comparison between the original post-gelation staining protocol and the modified version for pre-expansion-stained specimens presented in this publication (PS-ExM)

**Fig. 1.**
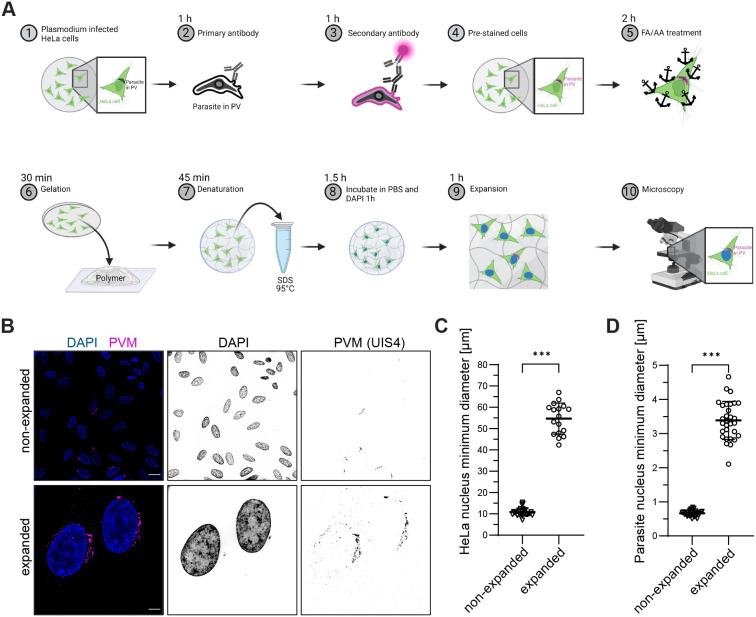
**PS-ExM workflow to study *Plasmodium* liver stage and expansion factor quantification.** (A) Overview of the procedure. Briefly, prior to the experiment, HeLa cells are seeded onto a coverslip and infected with sporozoites the next day (1). After fixation and permeabilisation, the cells are stained with primary (2) and secondary antibodies (3). The pre-stained cells (4) are subsequently fixed with formaldehyde (FA) and anchors are introduced with acrylamide (AA) (5), before the cells are embedded into a hydrogel (6) and the protein denatured in an SDS-containing buffer (7). The gel is washed with PBS before staining the nuclear DNA with DAPI (8). Cells are investigated with wide-field microscopy (10) after expanding the gel (9). Overview created with BioRender.com. (B) Expansion of infected HeLa cells at 6 hpi. DNA was stained with DAPI (blue) and the PVM (magenta) with UIS4 antibody staining. The expansion factor was determined based on DAPI staining. Scale bars: 20 µm (not adjusted for expansion where applicable). (C) Minimal nuclear diameter for non-expanded (*n*=27) and expanded (*n*=17) HeLa cells. The expansion factor of HeLa cell nuclei measured 5-fold. (D) Minimal nuclear diameter for non-expanded (*n*=30) and expanded (*n*=30) parasites. The expansion factor of parasite nuclei measured 5.1-fold. Data points are shown as individual values and mean±s.d. ***P<0.001 (two-tailed unpaired Student's *t*-test).

An overview of the PS-ExM protocol is depicted in Fig. 1A, and the detailed description can be found in the Materials and Methods section. An immunofluorescence assay was performed on infected HeLa cells. Primary and secondary antibody incubations were performed for 1 h. In comparison, in the original U-ExM protocol, each antibody incubation took 3 h (Table 1). The incubation time with formaldehyde and acrylamide was reduced to 2 h compared to the original U-ExM protocol, in which the incubation lasted for 5 h (Table 1). The addition of acrylamide results in functionalisation of the cells and subsequently allows to chemically link the proteins into a polyacrylamide gel matrix. Gelation was performed for 30 min in comparison to the 1.5 h in the original protocol (Table 1). For isotropic expansion, the cells were denatured for 30 min, which is three times shorter compared to the original protocol (Table 1). DNA of host cells and the parasites was stained with DAPI. After gel expansion, the cells were imaged. A major challenge in studying the *Plasmodium* liver stage in expansion microscopy is to be able to expand the intracellular parasite as well as the host cell under the same conditions. Representative images of non-expanded and expanded cells at 6 hours post-infection (hpi) are depicted in Fig. 1B. The parasitophorous vacuole membrane (PVM) was stained with anti-up-regulated in infective sporozoites gene 4 (UIS4) antibodies to identify infected HeLa cells. When comparing expanded and non-expanded cells, the morphological integrity of the PVM was preserved. The expansion factor measured was 5-fold for HeLa cell nuclei (Fig. 1C) and 5.1-fold for the parasite nuclei measured at 56 hpi (Fig. 1D). The PS-ExM protocol yielded isotropic expansion, as accessed through 3D sphericity measurements of parasite nuclei in both non-expanded and expanded samples (Fig. S1A,B). The isotropic expansion of the PS-ExM protocol was determined using the trophozoite developmental stage, as the parasite has a single nucleus with a spheric appearance at this stage (Roques et al., 2023). Furthermore, microtubule (α-tubulin) staining demonstrated the preservation of the cell morphology in the PS-ExM protocol (Fig. S1D). We compared the preservation of different epitopes between the original protocol and the adapted PS-ExM protocol – the epitopes of UIS4, lysosome-associated membrane protein 1 (LAMP1) and α-tubulin were adversely affected using the original protocol, whereas the PS-ExM protocol resulted in excellent preservation of epitopes (Fig. S1C,D).

### The pre-gelation staining expansion microscopy protocol allows for studying the interaction of *Plasmodium* liver stage parasites and host-cell lysosomes

**Fig. 2.**
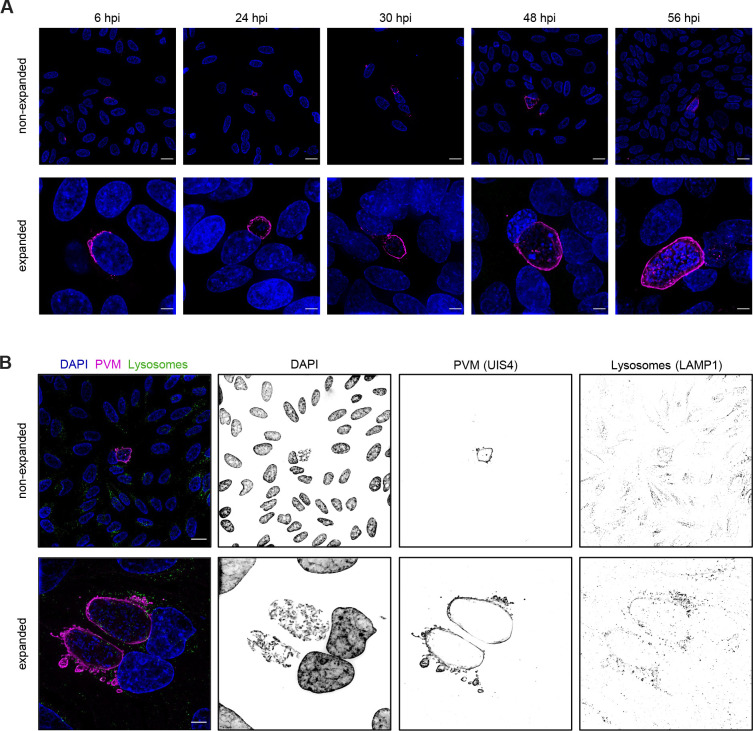
**PS-ExM of entire *Plasmodium* liver stage development.** (A) PS-ExM of entire *Plasmodium* liver stage development. Non-expanded (top row) and expanded (lower row) cells stained with DAPI to visualise nuclei (blue) and antibody staining of UIS4 to visualise the PVM (magenta) at different time points post infection (hpi). (B) Visualisation of parasite and host proteins in PS-ExM at 48 hpi. DNA DAPI staining is shown in blue. Antibody staining was performed to visualise the parasite protein UIS4, which stains the PVM (magenta) and LAMP1 staining host lysosomes (green). Images in this figure are representative of five repeats. Scale bars: 20 µm (not adjusted for expansion where applicable).

**Fig. 3.**
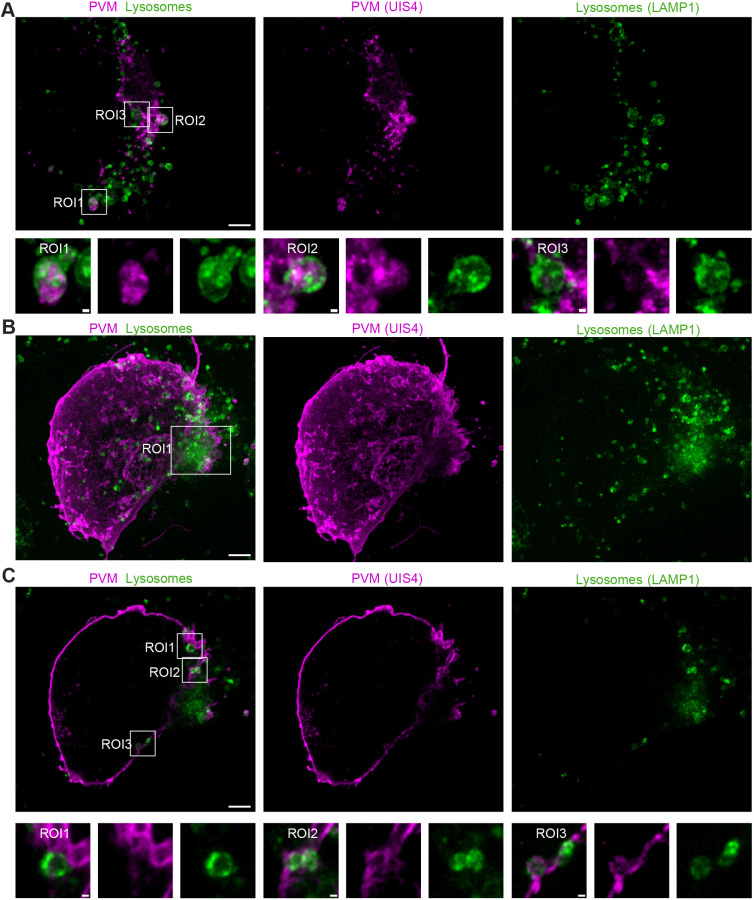
**PS-ExM sheds light on the interaction between parasite PVM and host lysosomes.** (A) Representative images of PS-ExM-expanded HeLa cells infected with *Plasmodium* sporozoites. The cells were PFA fixed at 6 hpi and stained with anti-LAMP1 (green) and anti-UIS4 (magenta). ROI1, ROI2 and ROI3 show how parts of the PVM were engulfed by lysosomal vesicles. (B,C) Images of expanded HeLa cells that were infected with *Plasmodium* sporozoites and fixed with PFA at 48 hpi. Lysosomes were stained with anti-LAMP1 (green) and the PVM with anti-UIS4 (magenta). (B) Maximum *Z*-projection of the confocal images. ROI1 shows how the host-cell lysosomes are accumulated in close proximity to the parasite tubo-vesicular network (TVN). (C) Single-plane image of the confocal images shown in B. ROI1–ROI3 show in detail how lysosomes get attached (ROI1), fused (ROI2), and become integrated into the parasite PVM (ROI3). All the images were acquired with the Nikon Ti 2 Crest V3 in spinning disc mode. Images in this figure are representative of triplicates. ROI, region of interest. Scale bars: 10 µm (main images); 1 µm (ROIs) (not adjusted for expansion).

During the *Plasmodium* liver stage, the parasite resides within a PV. The applicability of the PS-ExM was accessed for different phases of parasite liver stage development (Fig. 2A). The earliest time point was chosen as 6 hpi where only one single parasite nucleus is visible in the infected HeLa cell. The sporozoite, which initially has a banana-like shape, started becoming rounded at 24 hpi but still had a single nucleus. At 30 h post-infection, the parasite initiates the schizogony with massive replication, which is visible at 48 and 56 hpi as cells now show many parasite nuclei (Fig. 2A). Subsequently, the parasites undergo merogony and the PVM is ruptured. This time point was not included in this study given that the host cells detach from the coverslip at this phase. Besides being able to expand the whole *Plasmodium* liver stage, the applicability of the new PS-ExM to study host–pathogen interaction was tested using antibodies that detect proteins in the parasite and host cell. Previous studies have shown the association of lysosomes to the PV (da Silva et al., 2012; Niklaus et al., 2019). The interaction of lysosomes with the PVM was investigated in greater detail with PS-ExM. LAMP1, a lysosome membrane marker, was used to stain host-cell lysosomes and anti-UIS4 antibody staining was performed to outline the PVM. As previously described, lysosomes were found in close proximity to the PVM (Niklaus et al., 2019) (Fig. 2B). The lysosome interaction with the PVM was investigated at different time points of parasite development (24 hpi, 30 hpi and 48 hpi), as shown in Fig. S2A,B. By utilising Imaris software, we measured the fusion of lysosomes with the PVM, revealing that 25% of host lysosomes were attached to the PVM in non-expanded cells, whereas this number increased to 35% in expanded cells (Fig. S2C,D). We hypothesise that the improved staining achieved with the PS-ExM protocol contributes to this observed difference. In the PS-ExM samples, lysosomes appeared as vesicle-like structures, allowing for more accurate identification and quantification of lysosomes on the PVM compared to non-expanded controls, where lysosomes appeared as dots. The vesicle-like morphology of lysosomes provided valuable insights into the interaction between host-cell lysosomes and the PVM (Fig. 3). We observed that the lysosome sizes varied at the early time point of 6 hpi (Fig. 3A). The host cells might attack the pathogen by engulfing parts of the parasite PVM into lysosomes for digestion. Imaging showed that some large lysosomes contain PVM structures (Fig. 3A, ROI1 and ROI2). At the contact sides of lysosomes with the PVM, we observed a weaker UIS4 signal, suggesting fusion of the two membranes and their components (Fig. 3A, ROI3). Once the parasite became established within the host cell at 48 h post-infection, we observed a diffuse LAMP1 signal on the PVM (Fig. 3B, ROI1), again suggesting lysosome fusion with the PVM as previously described (Niklaus et al., 2019). Furthermore, we observed lysosomes that were attached or fused to the PVM (Fig. 3C, ROI1–ROI3). The fusion of the host lysosomes with the PVM resulted in a weaker UIS4 signal at the attachment point (Fig. 3C, ROI3) as observed at earlier time points of infection (Fig. 3A, ROI3).

### The PS-ExM protocol effectively retains the integrity of mitochondrial networks

**Fig. 4.**
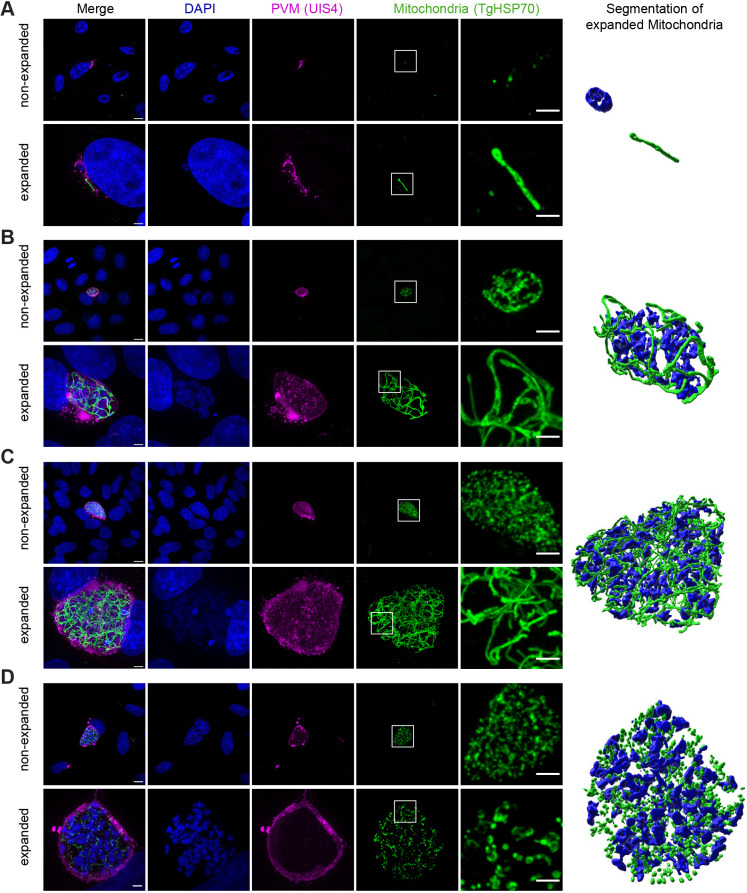
**PS-ExM of *Plasmodium* liver stage parasites allows visualisation of parasite mitochondria.** (A–C) Confocal images of parasite mitochondria in infected HeLa cells fixed with PFA at 6, 48 and 56 hpi, respectively. DNA was stained with DAPI and is shown in blue. Antibody staining was performed to visualise the PVM (UIS4, magenta) and parasite mitochondria (HSP70, green). Non-expanded samples are in the top row, and pre-gelation staining expansion microscopy images are at the bottom. The mitochondrial network and parasite nuclei were segmented with Imaris software. Images in this figure are representative of triplicates. Scale bars: 10 µm (main images); 5 µm (ROIs) (not adjusted for expansion where applicable).

To further evaluate the utility of the novel PS-ExM protocol in enhancing our understanding of parasite biology, we examined the preservation of mitochondria following expansion (Fig. 4). *Plasmodium* mitochondria were labelled with antibodies against *Toxoplasma gondii* heat shock protein 70 (TgHSP70), which also cross-reacts with *P. berghei* mitochondrial heat shock protein 70 (Eickel et al., 2013). We investigated mitochondrial development during the *Plasmodium* liver stage at various time points post-infection (6 hpi, 30 hpi, 48 hpi and 56 hpi). Remarkably, the expansion process did not compromise the structural integrity of the mitochondrial network of the parasite (Fig. 4; Movie 1). Utilising the TgHSP70 signal, we generated a 3D model of the expanded mitochondrial network using Imaris software (Fig. 4). The mitochondria of the parasite could be seen to undergo extensive branching following hepatocyte invasion, ultimately distributing within the forming merozoitesm as previously described (Stanway et al., 2011).

## DISCUSSION

In this study, U-ExM was used for the first time to investigate *Plasmodium* liver stage parasites. The protocol originally developed by Gambarotto et al. was adapted to be used on pre-stained *Plasmodium*-infected HeLa cells (Gambarotto et al., 2019, 2021). Although we used the same chemical approach as in the U-ExM protocol, we performed a pre-expansion staining ExM (PS-ExM). Importantly, the time required for the U-ExM protocol was approximately halved from 1 to 2 days of work. In the original protocol developed by Gambarotto et al., the antibody incubation times were rather long, with 3 hs per incubation (Gambarotto et al., 2021). Performing the staining on cells prior to gelation allows for reducing the incubation time to 1 hour. Furthermore, non-expanded controls can be performed simultaneously using the same staining protocol as for the expanded samples.

With the new PS-ExM protocol, both the host cell and the developing parasite exhibited an isotropic 5-fold expansion throughout the entire *Plasmodium* liver stage (Fig. 1). We evaluated the effectiveness of the PS-ExM protocol in preserving epitopes by staining the parasite vacuole membrane (UIS4), host lysosomes (LAMP1), and parasite mitochondria (TgHSP70). In comparison to the original protocol, the PS-ExM protocol demonstrated superior preservation of the UIS4 and LAMP1 epitopes (Fig. S1C,D). Additionally, the structural integrity of the mitochondrial networks of the parasite was maintained throughout the liver stage expansion, as depicted in Fig. 4. In summary, the PS-ExM protocol provides excellent preservation of parasite mitochondria, the PVM, and host lysosomes, making it a valuable tool for in-depth organelle studies. By using α-tubulin to delineate single-cell borders for quantifying lysosome attachment to the PVM, we also observed that the PS-ExM protocol better preserved microtubules compared to the original protocol (Fig. S1D). These results suggest that, as compared to the original protocol, the PS-ExM maintains epitopes more effectively. This might be due to epitope loss during the denaturation time in the U-ExM protocol, which has been previously observed for some epitopes (Chozinski et al., 2016; Gao et al., 2017; Tillberg et al., 2016). By performing the antibody staining at the beginning of the protocol, this problem was solved. Additionally, as previously described, the antibodies themselves are linked to the swellable hydrogel during the acrylamide treatment potentially enhancing the antibody signal (Gao et al., 2017).

Additionally, PS-ExM was used to investigate the host–pathogen interaction of lysosomes on the PVM. The lysosomes were seen as vesicle-like structures, and further details of the interaction between lysosomes and the parasite PVM were observed using the PS-ExM protocol (Figs 2 and 3). Therefore, this novel protocol might be a helpful tool for further research of the molecular processes underlying the fusion of lysosomes to the parasite PVM.

However, expanding and imaging whole HeLa cells for quantitative purposes results in huge data sets (after deconvolution, a single image was ∼10 GB for wide-field images, and for every experiment, at least ten images were acquired). Here, data storage and processing must be considered. Taken together, the PS-ExM protocol is faster compared to the original protocol, and it might be better suited for some epitopes that are otherwise lost during denaturation. The protocol here described can be further used to study host–pathogen interactions and gain further insight into the *Plasmodium* liver stage.

## MATERIALS AND METHODS

### Ethics statement

Mice were obtained from Harlan Laboratories or Charles River. All mice (C57BL/6 and BALB/c) were maintained and bred in the central animal facility of the University of Bern. Studies were strictly performed under the guidelines and laws of the Animal Research Ethics Committee of the Canton of Bern, Switzerland (Permit Number: BE98/19) and the University of Bern Animal Care and Use Committee Switzerland. The mice were between 6 and 26 weeks old.

### Mosquito maintenance

Mosquitoes (*Anopheles stephensi*) were maintained inside stock cages in the insectarium at the Institute of Cell Biology of the University of Bern. Briefly, mosquitoes were fed with blood by placing a Petri dish closed with parafilm containing the blood upside-down onto the mosquito cage. Blood was kept warm with a small Erlenmeyer containing hot water that was put on top of the Petri dish. The mosquitoes were allowed to feed for 30 to 40 min before the Petri dish containing the blood was removed. At 2 days after the feed, a beaker with water and filter paper was added to the mosquito cage to collect the mosquito eggs. The next day, the eggs were collected, washed once with 70% ethanol, and twice with water before being added to a metal bowl with a drop of about 10 µl of NobilFluid Artemia (JBL 33801 Germany), where they developed into larvae. After 2 days in the metal bowl, the larvae were transferred to a water-containing plastic bowl and fed with grounded Tetra TabiMin complete food tablets (Olibetta, 400080, Germany). After 7 days, the first pupae were collected and put in a stock cage, where they were kept at 27°C and 80% humidity until hatching. Adult mosquitoes were fed with 8% fructose supplemented with 0.2% PABA (Sigma-Aldrich, Cat. No. 100536). After 2 days, the female *A. stephensi* mosquitoes were ready to be infected with *P. berghei* parasites.

### Parasite maintenance in mosquitoes

A blood stabilate of the *P. berghei* ANKA strain expressing cytosolic mCherry under the Hsp70 promoter (Burda et al., 2015) was injected intraperitoneally into a naïve Balb/c mouse. When the mouse reached a parasitemia of 3 to 5%, 50 µl of its infected blood was diluted with 150 µl 1× phosphate-buffered saline (PBS) and injected intravenously into a new mouse that was intraperitoneally injected with 200 µl phenylhydrazine (6 mg ml^−1^ in PBS, Sigma-Aldrich, 114715) 3 days prior to infection. After 3 days, 5 µl of blood was collected from the mouse tail and diluted in 500 µl of 1× PBS to check the parasitemia with fluorescence-activated cell sorting (FACS). When the mouse reached a parasitemia of 5% with at least 0.5% to 1% of gametocytes, the mouse was anaesthetised and used to feed 100–150 female *Anopheles stephensi* mosquitoes for 1 h. Infected mosquitoes were kept at 20.5°C and 80% humidity and fed with 8% fructose containing 0.2% PABA for 16 to 26 days for their salivary gland dissection and mammalian HeLa cell infection.

### Mammalian cell culture and infection

Human epithelial HeLa cells (European Cell Culture Collection) were maintained at 37°C with 5% CO_2_. They were grown in minimum essential medium (MEM, BioConcept, 1-31F01-I) supplemented with Earle's salts and 10% heat-inactivated fetal calf serum (FCS, GE Healthcare) referred to as complete culture MEM. Medium contained 1% L-glutamine (Bioconcept, 5-10K00-H) and 1% penicillin-streptomycin (Bioconcept, 4-01F00-H. Cells were split every 4 days by treatment with accutase (Sigma-Aldrich, A6964). 40,000 HeLa cells were seeded on a coverslip in a 24-well plate (Greiner Bio-one, 662160) containing complete culture MEM. The next day, when the infected female *A.s stephensi* mosquitoes were between days 16 and 26 post-infection, the mosquitoes were anaesthetised with chloroform vapour (Merck, 67663 Germany) for ∼20 s. Anaesthetised mosquitoes were briefly dipped into 70% ethanol and then transferred to 1× PBS. The mosquitoes were then put into 200 μl of non-supplemented MEM medium, referred to as infection medium, on a slide, and the head was then meticulously removed using surgical forceps. The two sets of each of the three salivary gland lobes were isolated from the head and transferred into an Eppendorf tube containing 20 μl of infection medium. The salivary glands that had been dissected were kept on ice until infection. The sporozoite parasites were mechanically released from the salivary glands using a pestle (Sigma-Aldrich, Z359947) that was powered by a cordless motor (Sigma-Aldrich, Z359971) with 10 pulses of 1–2 s. To determine the number of sporozoites, 10 μl of the infection medium was introduced to a Neubauer chamber. The seeded HeLa cells were then infected by the addition of 20,000 sporozoites in 200 μl. The 24-well plate was spun at 1000 ***g*** for 1 min to allow the sporozoites to settle quickly before being incubated at 37°C with 5% CO_2_ for 2 h. To get rid of mosquito salivary gland debris and sporozoites that failed to infect HeLa cells, cells were washed twice with 500 μl of pre-warmed complete culture medium. Following that, cells were then incubated with 1 ml of complete culture medium at 37°C with 5% CO_2_ as previously described (Kaiser et al., 2017). The medium was exchanged with the cells at 24 and 48 hpi. At specific time points after infection (6 hpi, 24 hpi, 30 hpi, 48 hpi and 56 hpi), the cells were fixed and kept at 4°C for the immunofluorescence assay.

### Immunofluorescence assay

Infected HeLa cells were fixed with 4% paraformaldehyde (PFA) in PBS for 10 min at room temperature and subsequently washed twice with 1× PBS. Cells were permeabilised with 0.05% Triton X-100 (Fluka Chemie, T8787) for 5 min at room temperature, supplemented with 0.1% saponin (for washing out cytoplasmic background when staining lysosomes). Nonspecific antibody binding was reduced by blocking with 10% FCS in PBS for 20 min at room temperature. Subsequently, the cells were incubated with primary antibodies diluted in 10% FCS for 1 h at room temperature. The primary antibodies used were: anti-UIS4 rabbit (P-Sinnis, Baltimore, 1:1000 in PS-ExM and 1:500 in post-staining ExM), chicken anti-UIS4 (produced by Proteogenix in 2021, 1:1000), mouse anti-LAMP1 (Developmental Hybridoma Bank, clone H4A3, 1:1000 in PS-ExM and 1:500 in post-staining ExM), rabbit anti-TgHSP70 (gift from Dominique Soldati Favre, 1:500) and guinea pig anti-α-tubulin (Geneva Antibody Facility, AA345, 1:200 in PS-ExM and 1:125 in post-staining ExM) antibodies. The cells were then washed three times for 5 min each with 1× PBS and incubated with secondary antibodies at room temperature for 1 h, protected from light. The secondary antibodies used were: anti-rabbit-IgG conjugated to ATTO 647 (Sigma, 40839, 1:1000 in PS-ExM and 1: 500 in post-staining ExM), anti-mouse-IgG conjugated to Alexa Fluor 488 (Invitrogen Molecular Probes, A11001, 1:1000 in PS-ExM and 1:500 in post-staining ExM), anti-guinea pig-IgG conjugated to Alexa Fluor 594 (Invitrogen Molecular Probes, A11076, 1:1000 in PS-ExM and 1:500 in post-staining-ExM) and anti-chicken-IgY conjugated to Alexa Fluor 594 (Invitrogen Molecular Probes, A11042, 1: 500). All antibodies are additionally listed in Table S1. For non-expanded samples, the coverslips were stained with DAPI (Sigma, D9542, 1:100) for 5 min before being mounted with 5 μl ProLong™ Gold Antifade Mountant (Invitrogen, P36930). Images were acquired as described below.

### PS-ExM

The chemical approach used was based on the U-ExM protocol published by Gambarotto et al. (Gambarotto et al., 2019, 2021). The protocol was optimised to make it applicable for pre-gelation-stained samples. The incubation times were shortened to better preserve the fluorophores of the pre-stained sample. Infected HeLa cells were stained as described above and samples were kept protected from light until imaging. Cells were incubated in PBS containing 0.7% formaldehyde (FA, 36.5–38%, F8775, Sigma) and 1% acrylamide (AA, 40%, A4058, Sigma). The latter will link the cells to the polymer during gelation. Compared to the original U-ExM protocol (5 h incubation), the formaldehyde and acrylamide incubation time was shortened to 1.5 h at 37°C. Subsequently, gelation was performed as described by Gambarotto et al. Gelation solution was prepared freshly: 0.5% ammonium persulfate (APS, 17874, Thermo Fisher Scientific) and 0.5% tetramethylethylenediamine (TEMED, 17919, Thermo Fisher Scientific) were added to the monomer solution containing 19% sodium acrylate (SA, 97–99%, 408220, SIGMA), 10% acrylamide (AA, 40%, A4058, Sigma) and 0.1% N,N′-methylenebisacylamide (BIS, 2%, M1533, Sigma). Per cover slip, 35 µl gelation solution was used. After incubating the sample on ice for 5 min, the gelation was carried out for 30 min at 37°C. Subsequently, gels were incubated in 1 ml denaturation buffer (200 mM SDS, 200 mM NaCl and 50 mM Tris-HCl in deionised water, pH 9) with gentle agitation at room temperature for 15 min to detach the gel from the coverslip. Denaturation was carried out at 95°C in denaturation buffer for 30 min. Gels were briefly incubated in PBS and afterwards, the DNA was stained with 5 µg ml^−1^ DAPI (D9542-5MG, Sigma) diluted in 2% bovine serum albumin (BSA) at 37°C with gentle agitation for 1 h. Samples were expanded in deionised water for 1 h. If the sample was imaged a day after expansion, the gel was incubated in 0.2% propyl gallate (02370, Sigma) to avoid photobleaching of samples until imaging. The chemicals are additionally listed in Table S2.

### Mounting and image acquisition

After the final expansion in deionised water, the gels were cut and mounted on poly-D-lysine (A38904, Gibco) functionalised 35 mm glass-bottom dishes (D35-20-1.5-N, Cellvis, 35 mm glass bottom dish with 20 mm micro-well #1.5 cover glass). These closed sample holders strongly reduced evaporation and shrinkage of the gels. All images were acquired with a 60× oil objective (NA=1.4). The NIKON Ti 2 CREST V3 equipped with a Hamamatsu Flash 4.0 camera and a celesta light engine was used in widefield or spinning disc. Images were acquired with a *Z*-step size of 300 nm and pixel size of 108 nm. Images acquired in wide-field mode were deconvolved with Huygens Professional version (Huygens Remote Manager v3.8; Scientific Volume Imaging, The Netherlands, http://svi.nl).

### Image analysis

The open-source ImageJ platform was used to measure cell nuclei to determine the expansion factor (Schindelin et al., 2012). To measure parasite expansion, parasite nuclei were measured at 56 hpi. In comparison, a minimal HeLa cell diameter was determined at different time points after infection. The expansion factor was calculated by dividing the average nuclear diameter of expanded cells by the average nuclear diameter of non-expanded samples. The 3D and 4D image analysis software Imaris (Imaris X64, 9.9.0; Mar 11, 2022) was used to segment parasite mitochondria and nuclei (Fig. 3) and the lysosome attachment to the parasite PVM (Fig. S2). Briefly, to measure the number of lysosomes attached to the parasite PVM in infected HeLa cells, the PVM (stained with anti-UIS4) was used to generate the isosurface, and the lysosomal vesicles (stained with LAMP1) were used to generate isospots. The generation of the isosurface from the anti-UIS4 staining was carried out using an isosurface detail value of 0.215 for non-expanded and expanded PVM in conjunction with the absolute intensity. The lysosome isospots were derived in the ‘local contrast’ mode using the estimated point spread function (PSF). For non-expanded lysosomes, an estimated *XY*-diameter of 0.4 μm and an estimated *Z*-diameter of 0.5 μm were used, whereas for expanded lysosomes, an estimated *XY*-diameter of 1.5 μm and 2.4 μm of estimated *Z*-diameter were used. The isospots attached to the PVM were classified using the ‘Shortest Distance to Surfaces’ parameter, with the threshold set at 0 μm distance to the PVM. The isospots within infected HeLa cells were manually selected based on anti-α-tubulin staining, which was used to delimitate the single-cell border. The isospots of non-infected HeLa cells were excluded from the analysis. Isospot numbers as well as the shortest distance to surfaces and isosurface data were exported into a Microsoft Excel file. The proportion of LAMP1 isospots attached to the PVM was calculated by dividing the number of LAMP1 isospots attached to the PVM by the total number of LAMP1 isospots within the infected cell. The Imaris software was also used to compute the isosurface of parasite nuclei in acquired *Z*-stack images in a similar manner to the PVM isosurface details to determine isotropic expansion by measuring the sphericity of the nuclei in non-expanded and expanded samples. The data were structured and analysed using GraphPad Prism version 9.0.0 for Mac, GraphPad Software, San Diego, California, USA (www.graphpad.com). Statistical analysis was performed using a two-tailed unpaired Student's *t*-test and are denoted as ***P<0.001; **P<0.01; *P<0.05.

## Supplementary Material

10.1242/joces.261377_sup1 Supplementary information

## References

[JCS261377C1] Amino, R., Thiberge, S., Martin, B., Celli, S., Shorte, S., Frischknecht, F. and Ménard, R. (2006). Quantitative imaging of Plasmodium transmission from mosquito to mammal. *Nat. Med.* 12, 220-224. 10.1038/nm1350 16429144

[JCS261377C2] Beier, J. C. (1998). Malaria parasite development in mosquitoes. *Annu. Rev. Entomol.* 43, 519-543. 10.1146/annurev.ento.43.1.519 9444756

[JCS261377C3] Burda, P.-C., Roelli, M. A., Schaffner, M., Khan, S. M., Janse, C. J. and Heussler, V. T. (2015). A plasmodium phospholipase is involved in disruption of the liver stage parasitophorous vacuole membrane. *PLoS Pathog.* 11, e1004760. 10.1371/journal.ppat.1004760 25786000 PMC4364735

[JCS261377C4] Chen, F., Tillberg, P. W. and Boyden, E. S. (2015). Expansion microscopy. *Science* 347, 543-548. 10.1126/science.1260088 25592419 PMC4312537

[JCS261377C5] Chozinski, T. J., Halpern, A. R., Okawa, H., Kim, H.-J., Tremel, G. J., Wong, R. O. L. and Vaughan, J. C. (2016). Expansion microscopy with conventional antibodies and fluorescent proteins. *Nat. Methods* 13, 485-488. 10.1038/nmeth.3833 27064647 PMC4929147

[JCS261377C6] Cowman, A. F. and Crabb, B. S. (2006). Invasion of red blood cells by malaria parasites. *Cell* 124, 755-766. 10.1016/j.cell.2006.02.006 16497586

[JCS261377C7] da Silva, M., Thieleke-Matos, C., Cabrita-Santos, L., Ramalho, J. S., Wavre-Shapton, S. T., Futter, C. E., Barral, D. C. and Seabra, M. C. (2012). The host endocytic pathway is essential for plasmodium berghei late liver stage development. *Traffic* 13, 1351-1363. 10.1111/j.1600-0854.2012.01398.x 22780869

[JCS261377C8] Derbyshire, E. R., Mota, M. M. and Clardy, J. (2011). The next opportunity in anti-malaria drug discovery: the liver stage. *PLoS Pathog.* 7, e1002178. 10.1371/journal.ppat.1002178 21966266 PMC3178564

[JCS261377C31] Eickel, N., Kaiser, G., Prado, M., Burda, P. C., Roelli, M., Stanway, R. R. and Heussler, V. T. (2013). Features of autophagic cell death in Plasmodium liver-stage parasites. *Autophagy* 9, 568-580. 10.4161/auto.23689 23388496 PMC3627671

[JCS261377C9] Gambarotto, D., Zwettler, F. U., Le Guennec, M., Schmidt-Cernohorska, M., Fortun, D., Borgers, S., Heine, J., Schloetel, J.-G., Reuss, M., Unser, M. et al. (2019). Imaging cellular ultrastructures using expansion microscopy (U-ExM). *Nat. Methods* 16, 71-74. 10.1038/s41592-018-0238-1 30559430 PMC6314451

[JCS261377C10] Gambarotto, D., Hamel, V. and Guichard, P. (2021). Chapter 4 - Ultrastructure expansion microscopy (U-ExM). In *Methods in Cell Biology*, Vol. 161 (ed. P. Guichard and V. Hamel), pp. 57-81. Academic Press. 10.1016/bs.mcb.2020.05.006 33478697

[JCS261377C11] Gao, R., Asano, S. M. and Boyden, E. S. (2017). Q&A: Expansion microscopy. *BMC Biol.* 15, 50. 10.1186/s12915-017-0393-3 28629474 PMC5474863

[JCS261377C12] Heintzmann, R. and Ficz, G. (2006). Breaking the resolution limit in light microscopy. *Brief. Funct. Genomic.* 5, 289-301. 10.1093/bfgp/ell036 17170013

[JCS261377C13] Kaiser, G., De Niz, M., Burda, P.-C., Niklaus, L., Stanway, R. L. and Heussler, V. (2017). Generation of transgenic rodent malaria parasites by transfection of cell culture-derived merozoites. *Malar. J.* 16, 305. 10.1186/s12936-017-1949-y 28764716 PMC5540294

[JCS261377C14] Liffner, B. and Absalon, S. (2021). Expansion microscopy reveals plasmodium falciparum blood-stage parasites undergo anaphase with a chromatin bridge in the absence of mini-chromosome maintenance complex binding protein. *Microorganisms* 9, 2306. 10.3390/microorganisms9112306 34835432 PMC8620465

[JCS261377C15] Mota, M. M., Pradel, G., Vanderberg, J. P., Hafalla, J. C. R., Frevert, U., Nussenzweig, R. S., Nussenzweig, V. and Rodríguez, A. (2001). Migration of plasmodium sporozoites through cells before infection. *Science* 291, 141-144. 10.1126/science.291.5501.141 11141568

[JCS261377C16] Niklaus, L., Agop-Nersesian, C., Schmuckli-Maurer, J., Wacker, R., Grünig, V. and Heussler, V. T. (2019). Deciphering host lysosome-mediated elimination of Plasmodium berghei liver stage parasites. *Sci. Rep.* 9, 7967. 10.1038/s41598-019-44449-z 31138850 PMC6538699

[JCS261377C17] Plowe, C. V. (2022). Malaria chemoprevention and drug resistance: a review of the literature and policy implications. *Malar. J.* 21, 104. 10.1186/s12936-022-04115-8 35331231 PMC8943514

[JCS261377C18] Rankin, K. E., Graewe, S., Heussler, V. T. and Stanway, R. R. (2010). Imaging liver-stage malaria parasites. *Cell. Microbiol.* 12, 569-579. 10.1111/j.1462-5822.2010.01454.x 20180802

[JCS261377C19] Rashpa, R. and Brochet, M. (2022). Expansion microscopy of Plasmodium gametocytes reveals the molecular architecture of a bipartite microtubule organisation centre coordinating mitosis with axoneme assembly. *PLoS Pathog.* 18, e1010223. 10.1371/journal.ppat.1010223 35077503 PMC8789139

[JCS261377C20] Roques, M., Bindschedler, A., Beyeler, R. and Heussler, V. T. (2023). Same, same but different: Exploring Plasmodium cell division during liver stage development. *PLoS Pathog.* 19, e1011210. 10.1371/journal.ppat.1011210 36996035 PMC10062574

[JCS261377C21] Schindelin, J., Arganda-Carreras, I., Frise, E., Kaynig, V., Longair, M., Pietzsch, T., Preibisch, S., Rueden, C., Saalfeld, S., Schmid, B. et al. (2012). Fiji: an open-source platform for biological-image analysis. *Nat. Methods* 9, 676-682. 10.1038/nmeth.2019 22743772 PMC3855844

[JCS261377C22] Snow, R. W., Guerra, C. A., Noor, A. M., Myint, H. Y. and Hay, S. I. (2005). The global distribution of clinical episodes of Plasmodium falciparum malaria. *Nature* 434, 214-217. 10.1038/nature03342 15759000 PMC3128492

[JCS261377C30] Stanway, R.R., Mueller, N., Zobiak, B., Graewe, S., Froehlke, U., Zessin, P.J.M., Aepfelbacher, M. and Heussler, V.T. (2011). Organelle segregation into Plasmodium liver stage merozoites. *Cell. Microbiol.* 13, 1768-1782. 10.1111/j.1462-5822.2011.01657.x 21801293

[JCS261377C23] Tavares, J., Formaglio, P., Thiberge, S., Mordelet, E., Van Rooijen, N., Medvinsky, A., Ménard, R. and Amino, R. (2013). Role of host cell traversal by the malaria sporozoite during liver infection. *J. Exp. Med.* 210, 905-915. 10.1084/jem.20121130 23610126 PMC3646492

[JCS261377C24] Tillberg, P. W., Chen, F., Piatkevich, K. D., Zhao, Y., Yu, C.-C., English, B. P., Gao, L., Martorell, A., Suk, H.-J., Yoshida, F. et al. (2016). Protein-retention expansion microscopy of cells and tissues labeled using standard fluorescent proteins and antibodies. *Nat. Biotechnol.* 34, 987-992. 10.1038/nbt.3625 27376584 PMC5068827

[JCS261377C25] Tse, E. G., Korsik, M. and Todd, M. H. (2019). The past, present and future of anti-malarial medicines. *Malar. J.* 18, 93. 10.1186/s12936-019-2724-z 30902052 PMC6431062

[JCS261377C26] Vaughan, A. M. and Kappe, S. H. I. (2017). Malaria parasite liver infection and exoerythrocytic biology. *Cold Spring Harb. Perspect. Med.* 7, a025486. 10.1101/cshperspect.a025486 28242785 PMC5453383

[JCS261377C27] Venugopal, K., Hentzschel, F., Valkiūnas, G. and Marti, M. (2020). Plasmodium asexual growth and sexual development in the haematopoietic niche of the host. *Nat. Rev. Microbiol.* 18, 177-189. 10.1038/s41579-019-0306-2 31919479 PMC7223625

[JCS261377C28] White, N. J. (2004). Antimalarial drug resistance. *J. Clin. Invest.* 113, 1084-1092. 10.1172/JCI21682 15085184 PMC385418

