# Supplementary material for: Pre-gelation staining expansion microscopy for visualisation of the Plasmodium liver stage
Source: J Cell Sci. 2023 Nov 30;136(22):jcs261377. doi: 10.1242/jcs.261377 (PMC10729816; doi:10.1242/jcs.261377)
Supplement: Supplementary information [file joces-136-261377-s1.pdf]

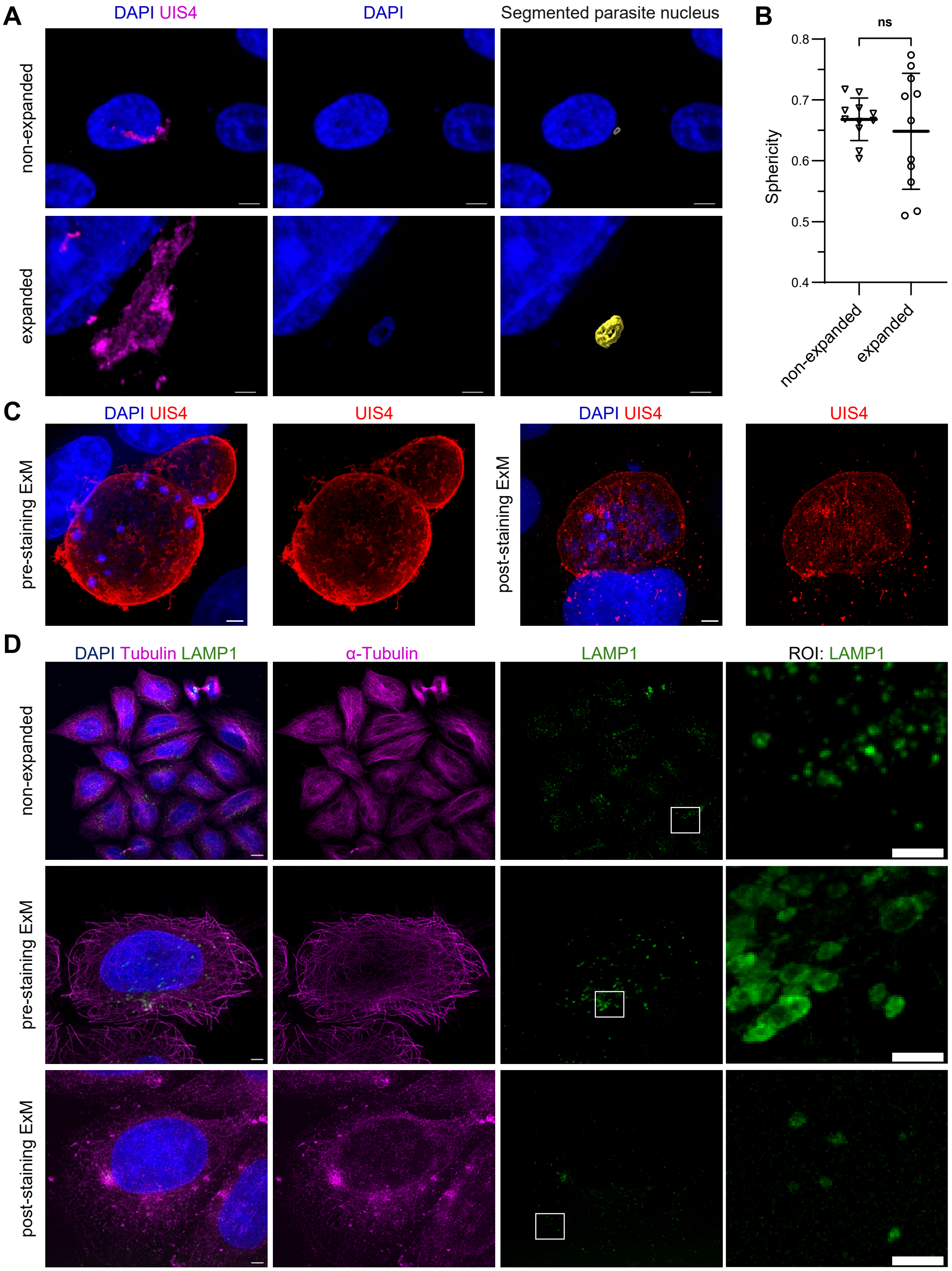

Journal of Cell Science • Supplementary information

Fig. S1. PS-ExM results in isotropic expansion and preservation of epitope. **(A)** z-stack confocal images of non-expanded and expanded fixed infected cells at 6 hpi. The PVM was stained with anti-UIS4 (magenta), and the host cell and parasite nuclei were stained with DAPI (blue). To determine the isotropicity of the PS-ExM protocol, the nucleus sphericity was measured using the 3D and 4D image analysis software Imaris. Briefly, the parasite DAPI signal was used to compute the 3D isosurface and measure the sphericity. A nucleus with an exact spherical shape will have a sphericity value of 1, which is the maximum. **(B)** Non-expanded (n = 11) and PS-ExM-expanded (n = 11) nuclei exhibit an average sphericity of 0.67 and 0.65, respectively, with no significant difference. Image scale bar = 5 µm. Data points are shown as individual values and mean ± SD. Statistical analysis was performed using the student's t-test: *** P <0.001; ** P <0.01; * P <0.05. **(C)** Comparison between PVM epitope preservation in the pre-staining ExM and the post-staining ExM protocols. The parasite PVM was stained with anti-UIS4 (red) and the parasite and HeLa cell nuclei with DAPI (blue). Image scale bars = 10 µm. **(D)** Comparison of epitope preservation in the non-expanded, pre-staining ExM, and post-staining ExM HeLa cells. The host cell's microtubules were stained with anti-α-tubulin (magenta), lysosomes with anti-LAMP1 (green), and cell nuclei with DAPI (blue). Image scale bar = 10 µm.

Journal of Cell Science • Supplementary information

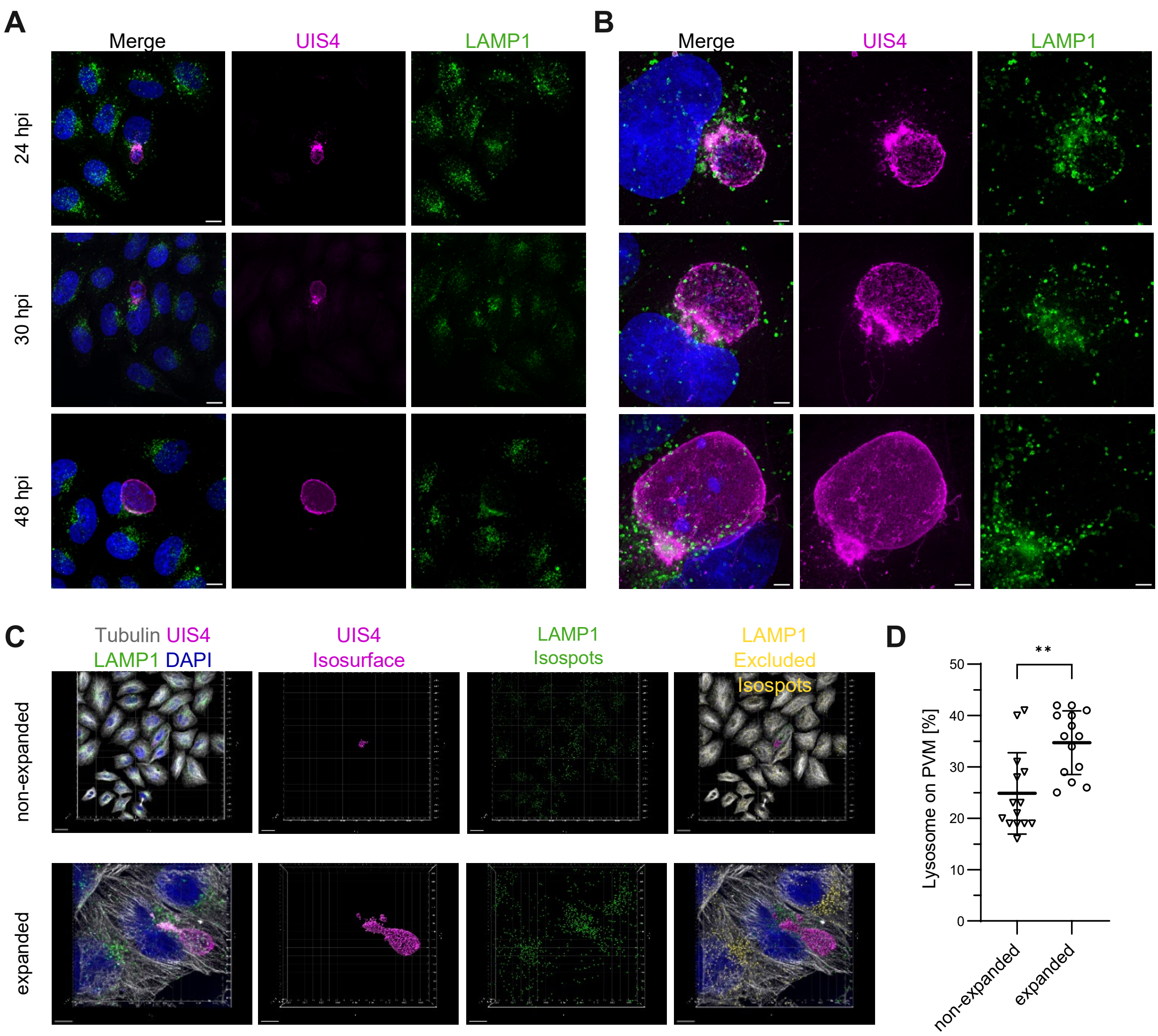

Fig. S2. PS-ExM improves the quantification of the host lysosome and parasite PVM interaction. (A and B) Confocal images of infected HeLa cells. The HeLa cells were infected with *Plasmodium* sporozoites and fixed at different time points (24 hpi, 30 hpi, and 48 hpi). Cells were stained with anti-LAMP1 (green), anti-UIS4 (magenta), and DAPI for the host cell and parasite nuclei (blue). The images show how the host cell lysosome interacts with the parasite during the liver stage development of the parasite until schizogony. Image scale bar =10 µm. **(A)** Non- expanded cells. **(B)** Expanded cells. **(C)** To quantify the lysosome-PVM interaction, the 3D and 4D image analysis software Imaris was used, as described in the method section. The 24 hpi cells were used for the quantification. Briefly, the anti-LAMP1 (green) signal was used to compute 3D lysosome isospots, and the anti-UIS4 signal (magenta) was used to compute the PVM 3D isosurface. The anti-α-tubulin signal (grey) was used to delimit a single cell border and exclude the LAMP1 signal of non-infected cells (excluded isospots in yellow). The images show non-expanded and expanded cells. Scale bar = 20 µm. **(D)** Percentage of attached lysosomes to the PVM per infected cell in non-expanded (n = 14) and expanded (n = 14) cells. On average, 35% of the host lysosome was fused to the PVM (0 µm distance to the PVM) in the expanded cells and 25% in the non-expanded cells. Data points are shown as individual values and mean ± SD. Statistical analysis was performed using the student's t-test: *** P <0.001; ** P <0.01; * P <0.05.

Journal of Cell Science • Supplementary information

Table S1. Overview of antibodies and chemicals used for the immunofluorescence assay.

Antibody/Chemical	Company/Provider	Catalogue Number	PS-ExM dilution	Post-staining ExM dilution
anti-UIS4 rabbit	P-Sinnis, Baltimore		1:1000	1:500
anti-UIS4 chicken	Produced by Proteogenix in 2021		1:1000	
anti-LAMP1 mouse	Developmental Hybridoma Bank, clone H4A3		1:1000	1:500
anti-TgHSP70 rabbit	Gift from Dominique Soldati Favre		1:500	
anti-α-tubulin guinea pig	Geneva Antibody Facility	AA345	1:200	1:125
anti-rabbit ATTO 647	SIGMA	40839	1:1000	1:500
anti-mouse Alexa Fluor 488	Invitrogen Molecular Probes	A11001	1:1000	1:500
anti-guinea pig Alexa Fluor 594	Invitrogen Molecular Probes	A11076	1:1000	1:500
anti-chicken Alexa Fluor 594	Invitrogen Molecular Probes	A11042	1:500	
TritonX 100	Fluka Chemie	T8787	0.05%	
ProLong^{TM} Gold antifade reagent	Invitrogen	P36930		
DAPI (100 µg ml^{-1})	SIGMA	D9542	1:100	

Journal of Cell Science • Supplementary information

Table S2. Overview of chemicals used for expansion microscopy: for each chemical, the abbreviation (abbr.), company, catalogue number, amount, storage and additional comments are given.

Chemical	Abbr.	Company	Catalogue Number	Amount	Comments	Storage
Formaldehyde 36.5–38%	FA	SIGMA	F8775	25 ml	TOXIC! Ready to use. Use under the hood.	RT
Acrylamide 40%	AA	SIGMA	A4058	100 ml	TOXIC! Ready to use. Use under the hood.	4°C
N,N-methylene-bisacrylamide 2%	BIS	SIGMA	M1533	25 ml	TOXIC! Ready to use. Use under the hood.	4°C
Sodium Acrylate	SA	SIGMA	408220	25 g	TOXIC! Use powder under the hood.	powder at -20°C, 38% solution at 4°C in the dark!
Ammonium persulfate	APS	Thermo-Fisher	17874		Make 10% aliquots (à 20 µl)	aliquots -20°C
Tetramethyl-ethylene-diamine	TEMED	Thermo-Fisher	17919		Make 10% aliquots (à 20 µl)	aliquots -20°C
Poly-D-Lysine		Gibco	A38904	100 ml	Ready to use	4°C
4′,6-diamidino-2-phenylindole	DAPI	SIGMA	D9542	5 mg	Aliquots with 5 mg ml^{-1} (à 10µl)	stock and aliquots at -20°C in the dark
Propyl gallate		SIGMA	02370		Powder	RT

Journal of Cell Science • Supplementary information

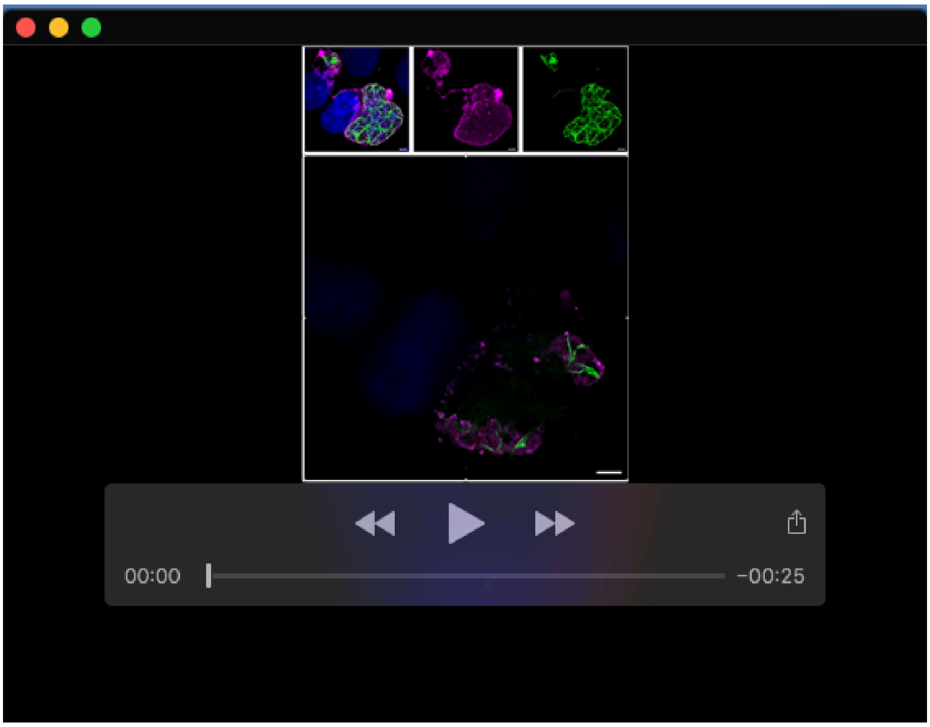

Movie 1. PS-ExM of the parasite mitochondrial network stained with anti-TgHSP70 (green), PVM stained with anti-US4 (magenta), and nuclei stained with DAPI (blue). Scale bars: 10 μm.
